# High prevalence of altered cardiac repolarization in patients with COPD

**DOI:** 10.1186/1471-2466-14-55

**Published:** 2014-04-02

**Authors:** Noriane A Sievi, Christian F Clarenbach, Giovanni Camen, Valentina A Rossi, Arnoldus JR van Gestel, Malcolm Kohler

**Affiliations:** 1 Pulmonary Division, University Hospital Zurich, Zurich, Switzerland; 2 Institute of Human Movement Sciences and Sport, ETH Zurich, Zurich, Switzerland; 3 Zurich Centre for Integrative Human Physiology, University of Zurich, Zurich, Switzerland; 4 Chair Respiratory Medicine, Clinical Director Division of Pulmonology, University Hospital Zurich, Zurich, Switzerland

## Abstract

**Background:**

Altered cardiac repolarization and increased dispersion of repolarization have been identified as risk factors for sudden cardiac death (SCD). The prevalence of and the mechanisms contributing to altered cardiac repolarization are currently unknown in COPD.

**Methods:**

In 91 COPD patients, 32 controls matched for age, cardiovascular risk and medication, and 41 healthy subjects, measures of cardiac repolarization and dispersion of repolarization (QTc interval, QT dispersion) were derived from 12-lead electrocardiography (ECG). Prevalence rates of heart rate corrected QT (QTc) >450ms and QT dispersion >60ms were determined to assess the number of subjects at risk for SCD. Univariate and multivariate analyses were used to identify possible factors contributing to altered cardiac repolarization.

**Results:**

QTc was found to be prolonged in 31.9% and QT dispersion in 24.2% of the COPD patients compared to 12.5% in matched controls and 0% in healthy subjects. The QTc interval was longer in COPD patients compared to matched and healthy controls respectively (437.9 ± 29.5 vs. 420.1 ± 25.3 ms, p = 0.001 and vs. 413.4 ± 18.2 ms, p < 0.001). QT dispersion was significantly increased in COPD patients compared to healthy subjects (45.4 (34.8 , 59.5) vs. 39.7 (29.3 , 54.8) ms, p = 0.049). Only oxygen saturation was independently associated with QTc duration in multivariate analysis (β = -0.29, p = 0.015).

**Conclusion:**

One third of a typical COPD population has altered cardiac repolarization and increased dispersion of repolarization, which may be related to hypoxia. Altered cardiac repolarization may expose these patients to an increased risk for malignant ventricular arrhythmias and SCD.

## Background

Chronic obstructive pulmonary disease (COPD) is associated with an increased risk of cardiovascular morbidity and mortality [1-3]. Previous population-based studies suggested that patients with COPD have a two to three fold increased risk of sudden cardiac death (SCD) [4]. However, the mechanisms underlying the association between COPD and SCD are currently unclear and predictors of malignant cardiac arrhythmias and SCD in COPD have not been defined.

Alteration of cardiac repolarization is an important mechanism for the development of malignant arrhythmias and the occurrence of SCD [5-7]. Measures derived from the surface electrocardiography (ECG) represent the dispersion of repolarization and the electrical inhomogeneity of the ventricles during repolarization [7,8]. The findings of previous studies in patients with heart failure [9], patients with long QT syndrome [10] and elderly persons [11] indicated an association between alteration of measures of cardiac repolarization, such as QT interval and QT dispersion with the development of malignant arrhythmia and SCD.

Evidence from longitudinal studies suggests that a low forced expiratory volume in 1 s (FEV_1_) is associated with an increased risk for ischemic heart and cerebral disease and SCD, even after correcting for conventional cardiovascular risk factors [12,13]. It has also been suggested that COPD patients may have a higher frequency of cardiac arrhythmias and the severity of airflow obstruction seems to be associated with the occurrence of arrhythmia [14,15].

Preliminary evidence from a small case-control study suggests that increased maximal QT interval is associated with the development of ventricular arrhythmia in patients with COPD [16]. However, the prevalence of and the mechanisms contributing to altered cardiac repolarization in patients with COPD are currently unknown.

Therefore, the aim of this study was to evaluate the prevalence of and possible factors contributing to altered cardiac repolarization in a group of COPD patients.

## Methods

### Subjects

#### COPD patients

157 patients aged between 40 and 75 years with objectively confirmed COPD according to GOLD-guidelines [17] were assessed for eligibility in the pulmonary outpatient clinic at the University Hospital of Zurich, Switzerland between October 2009 and January 2013. Patients were excluded if they had suffered from a COPD exacerbation within the last 6 weeks or if they suffered from mental or physical disability precluding informed consent or compliance with the protocol.

#### Matched control subjects

The patient database of the Pulmonary Division of the University Hospital Zurich, Switzerland was screened for control subjects with normal lung function and matched for age, Pocock risk score use of beta blockers and medication affecting QT duration.

#### Healthy control subjects

Subjects were eligible for the study if they were between 18 and 75 years old and healthy. Exclusion criteria were a previous diagnosis of any lung disease, arterial hypertension, any aortic or cardiac disease.

The study was conducted in accordance with the declaration of Helsinki of the World Medical Association. The Ethics Committee of the Canton of Zurich approved the study (EK-ZH-NR: 1734) and all subjects gave written informed consent to participate when data were obtained for research purposes.

### Measurements

#### Cardiovascular risk and medication

The Pocock risk score was used to predict the individuals’ 5-year risk of death due to a cardiovascular cause [18]. The Pocock risk score is calculated from 11 known cardiovascular risk factors, including sex, age, smoking status, systolic blood pressure, cholesterol, creatinine, height, diabetes, previous myocardial infarction, stroke and left ventricular hypertrophy.

Patients and control subjects who were included in the study were asked if they used medications affecting the QT interval according to the International Registry of Drug-induced Arrhythmias maintained by the Georgetown University [19].

### Respiratory variables

All participants underwent standard pulmonary functional testing according to American Thoracic Society guidelines [20] to measure forced expiratory volume in one second (FEV_1_) and forced vital capacity (FVC).

Oxygen saturation (SaO_2_) was measured with a fingertip oximeter PC-60C after the participants rested 5min in supine position.

### Electrocardiography

**Figure 1:**
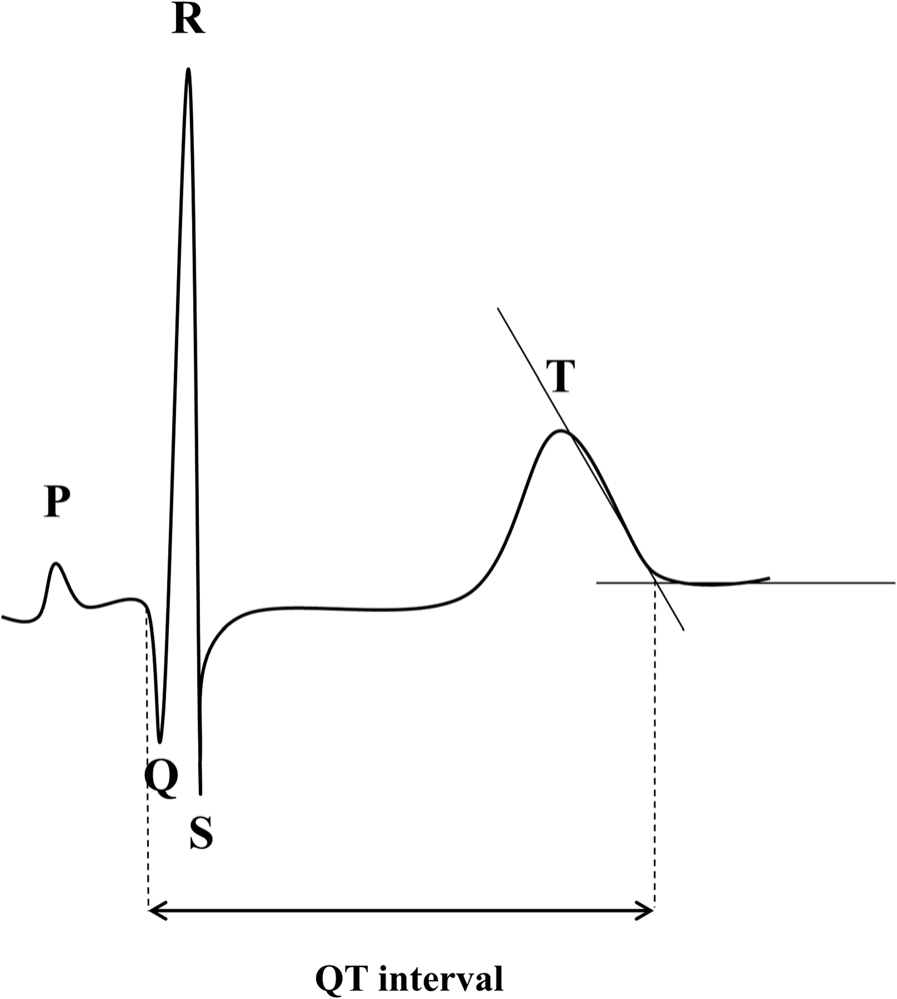
The length of the QT interval was obtained by identifying the QRS onset and the point at which the downward slope of the T wave returns to baseline.

Patients and control subjects were asked to abstain from alcohol, tobacco, or caffeine on the day the measurements were performed. Room temperature and lighting were set at the same level for all measurements. The patients rested for five minutes in supine position before measurements were performed. For all electrocardiographic recordings a commercially available 12-lead ECG (AT 104 PC, Schiller-Reomed AG, Switzerland) was used and set at 25-mm/s paper speed and 10-mm/mV amplitude. Measurements of the ECG intervals were performed in duplicate with dedicated ECG analysis software (DatInf® Measure 2.1d, DatInf GmbH, Tübingen, Germany) by two investigators who were blinded to the patient’s data as previously described [21]. The mean of the ECG interval measurements by the two investigators was calculated and used for statistical analysis. Four consecutive heart cycles were analysed for each lead. The mean value for each lead of the twelve leads was calculated. Measures of cardiac repolarization were determined as indicated in Figure 1. The QT interval was defined as the time from the earliest onset of the QRS complex to the end of the T wave [22]. The end of the T wave was defined as the cutting point of the tangent to the downward slope of the T-wave and the isoelectric line [23]. As a measurement of dispersion of repolarization, QT dispersion was defined as the difference between the lead with the maximal and the lead with the minimal QT interval duration [24].

QTc (QT interval corrected for heart rate) was corrected for heart rate by using the Bazett formula [25].

### Data analysis and statistics

All results are shown as mean values and standard deviation (SD) unless otherwise stated. Statistical analysis was performed with Statistica V6.0 (StatSoft, Tulsa, OK, USA) and STATA 12 (StataCorp, Texas, USA). Differences in QTc interval duration, cardiovascular risk factors, respiratory variables and use of medications between COPD patients, matched controls and healthy control subjects respectively were assessed by one-way ANOVA and χ^2^ tests. Kruskal Wallis test was used to compare QT dispersion between groups. Post hoc analysis was assessed for variables that showed a p-value of <0.1 across the three groups. For QTc, altered cardiac repolarization was defined as a value of >450ms and for QT dispersion of >60ms [16,26].

Univariate regression analysis was used to investigate associations between repolarization duration (dependent variable) and cardiovascular risk factors as well as respiratory variables and medications. Multivariate analysis involved regression of variables that showed a univariate regression p-value of <0.1 with adjustment for potential confounders (age). Residual analysis of the model was performed to check the regression assumptions. A two-sided p-value of <0.05 was considered to be statistically significant.

## Results

### Study participants

**Figure 2:**
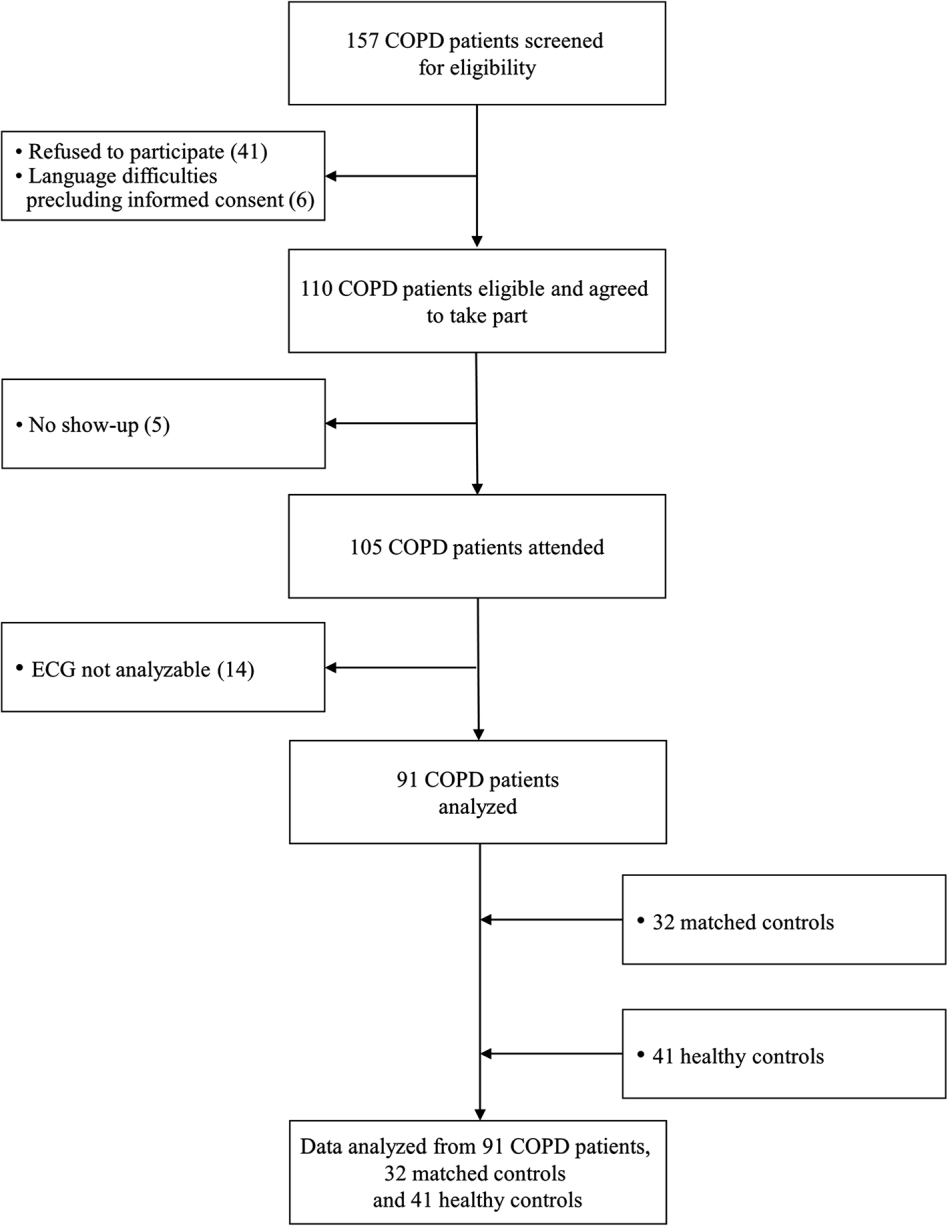
Study flow.

157 COPD patients were screened for eligibility for this study. 91 COPD patients (34% GOLD stage I/II, 27% III, 39% IV), 32 control subjects matched for age, cardiovascular risk and medication and 41 healthy subjects entered the final analysis (Figure 2). Patient characteristics are shown in Table 1.

**Table 1:** Participant characteristics

	**COPD N = 91**	**Matched controls N = 32**	**Healthy controls N = 41**
**Demographics and cardiovascular risk**			
Age, y	62 (7.1)^†^	62 (5.9)^¶^	30 (8.7)
Male/Female	61/30	24/8	24/17
BMI, kg/m^2^	27.9 (6.0)^†^	29.2 (4.4)^¶^	22.8 (3.1)
Current smokers, N (%)	19 (21)	7 (22)	5 (13)
Pack years of smoking, N	43 (23.7)*^†^	9 (18.2)	1 (5.1)
Arterial hypertension, N (%)	49 (54)^†^	23 (72)^¶^	0 (0)
CAD, N (%)	18 (20)^†^	3 (9)^¶^	0 (0)
Arrhythmia, N (%)	19 (21)	8 (25)	4 (10)
Diabetes, N (%)	13 (14)^†^	3 (9)^¶^	0 (0)
Cholesterol, mmol/l	5.2 (1.1)	4.9 (1.0)	5.1 (1.1)
Pocock risk score, %	2.2 (2.1)^†^	2.1 (0.9)^¶^	0.3 (0.3)
**Respiratory variables**			
FEV_1_% pred.	45 (22.4)*^†^	104 (16.0)	100 (11.1)
FVC% pred.	77 (18.3)*^†^	108 (14.0)	104 (8.2)
FEV_1_/FVC,%	45 (15.2)*^†^	78 (5.5)	80 (5.9)
SaO_2_, kPa	93.8 (3.4)^†^	95.1 (1.3)^¶^	98.0 (1.4)
**Medication**			
Medication affecting QT, N (%)	12 (13)^†^	4 (13)^¶^	0 (0)
Beta blockers, N (%)	18 (20)^†^	10 (31)^¶^	0 (0)
Antihypertensive medication, N (%)	47 (52)^†^	19 (59)^¶^	0 (0)
Anticholinergic medication, N (%)	62 (68)*^†^	0 (0)	0 (0)
Combined inhaled long-acting β-adrenergic and steroid, N (%)	53 (58)*^†^	0 (0)	0 (0)
Statins, N (%)	30 (33)^†^	11 (34)^¶^	0 (0)

Values are mean (SD). *p < 0.05 COPD vs. matched controls. ^†^p < 0.05 COPD vs. healthy controls. ^¶^p < 0.05 matched controls vs. healthy controls. BMI: body mass index; CAD: coronary artery disease; FEV_1_: forced expiration in one second; FVC: forced volume capacity; SaO_2_: oxygen saturation.

### Prevalence of altered repolarization measures

The QTc interval was significantly longer in COPD patients compared to matched controls (437.9 ± 29.5 vs. 420.1 ± 25.3 ms, p = 0.001) and compared to healthy subjects (437.9 ± 29.5 vs. 413.4 ± 18.2 ms, p < 0.001). QT dispersion showed a non-significant trend to be increased in COPD patients compared to matched controls (45.4 (34.8, 59.5) vs. 39.7 (29.3, 54.8) ms, p = 0.102). COPD patients showed a significantly increased QT dispersion compared to healthy subjects (45.4 (34.8, 59.5) vs. 40.8 (33.0, 48.8) ms, p = 0.049).

**Figure 3:**
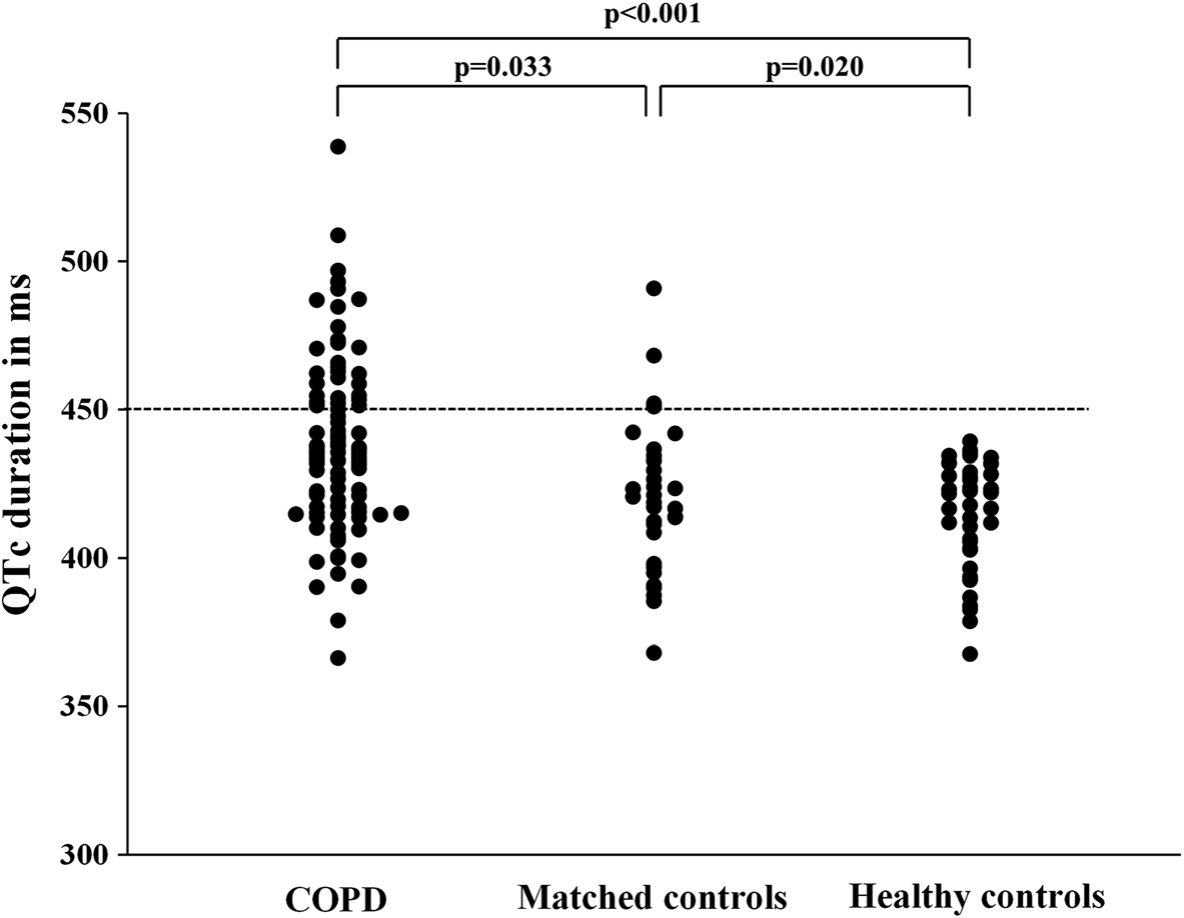
**The distribution of repolarization duration of QTc is illustrated.** Each point represents the repolarization duration of an individual subject. The dotted line marks the threshold indicating altered cardiac repolarization and thus risk for SCD.

Of 91 COPD patients, 31.9% showed a prolonged QTc interval >450ms compared to 12.5% in matched controls (p = 0.033) and 0% in healthy subjects (p < 0.001). 24.2% of the COPD patients had an increased QT dispersion >60 ms compared to 12.5% in matched controls (p = 0.164) and 0% in healthy subjects (p < 0.001) (Figure 3).

### Determinants of altered cardiac repolarization

#### Univariate regression analysis

QTc was associated with age, pack years of smoking, coronary artery disease (CAD), FEV_1_% pred., FVC% pred., SaO_2_, the use of combined inhaled long-acting β-adrenergic and steroid, anticholinergic medication and statins (Table 2). QT dispersion showed a significant association with pack years of smoking (β = 0.17, p = 0.033) and the use of statins (β = 0.16, p = 0.041).

**Table 2:** Univariate regression analysis of predictors for QTc in all participants

	**β**	**95% confidence interval**	**p-value**
**Cardiovascular risk**			
Age, y	0.30	0.15/0.45	<0.001*
Male/Female	-0.08	-0.23/0.08	0.317
BMI, kg/m^2^	0.13	-0.02/0.28	0.104
Current smoker, N (%)	-0.01	-0.16/0.15	0.934
Pack years of smoking, N	0.28	0.13/0.44	<0.001*
Pocock risk score, %	0.17	-0.00/0.35	0.054
**Respiratory variables**			
FEV_1_% pred.	-0.30	-0.48/-0.12	0.001*
FVC% pred.	-0.32	-0.50/-0.15	<0.001*
SaO_2,_ %	-0.34	-0.51/-0.17	<0.001*
**Medication**			
Medication affecting QT, N (%)	0.13	-0.03/0.28	0.108
Beta blocker, N (%)	0.05	-0.11/0.20	0.558
Combined inhaled long-acting β-adrenergic and steroid, N (%)	0.28	0.13/0.43	<0.001*
Statins, N (%)	0.23	0.07/0.38	0.004*

*p < 0.05; QTc: QT interval corrected for heart rate; BMI: body mass index; FEV_1_: forced expiration in one second; FVC: forced vital capacity; SaO_2_: arterial oxygen saturation.

#### Multivariate analysis

Table 3 shows a multiple regression model with QTc as dependent and age, gender, pack years of smoking, Pocock risk score, CAD, FEV_1_% pred., SaO_2_, use of combined inhaled long-acting β-adrenergic and steroid, use of anticholinergic medication and consumption of statins as independent variables. SaO_2_ was the only independently associated variable. In Multivariate analysis performed for QT dispersion as dependent and age, gender, BMI, pack years of smoking, SaO_2_, use of beta blocker, combined inhaled long-acting β-adrenergic and steroid and consumption of statins as independent variables, none of the variables showed an independent association.

**Table 3:** Multiple regression analysis of predictors for QTc in all participants

	**β**	**95% confidence interval**	**p-value**
Age, y	-0.04	-0.28/0.19	0.713
Pack years of smoking, N	-0.11	-0.33/0.12	0.349
Pocock risk score, %	0.07	-0.17/0.30	0.587
FEV_1_% pred.	-0.16	-0.42/0.11	0.244
SaO_2,_%	-0.29	-0.51/-0.07	0.011*
Combined inhaled long-acting β- adrenergic and steroid, N (%)	0.11	-0.13/0.35	0.375
Statins, N (%)	0.08	-0.12/0.29	0.426

*p < 0.05; QTc: QT interval corrected for heart rate; FEV_1_: forced expiration in one second; SaO_2_: arterial oxygen saturation.

## Discussion

This study investigated the prevalence of altered cardiac repolarization in a heterogeneous group of COPD patients and evaluated possible underlying risk factors. The main findings of this study are that approximately one third of a typical COPD population has altered cardiac repolarization and increased dispersion of repolarization, which may be related to hypoxia. Altered cardiac repolarization may expose these patients to an increased risk for malignant ventricular arrhythmias and SCD.

Several pathologies have been reported to increase the risk of SCD including CAD, cardiomyopathies, congenital heart disease and electrophysiological abnormalities including long QT syndrome [27].

A prolonged QT interval and increased QT dispersion are markers of an increased risk for malignant cardiac arrhythmia and SCD [9,26]. The QT interval represents the electrocardiographic correlate of ventricular de- and repolarization including the vulnerable period for reentry tachycardia. As such, QTc prolongation to >450 ms has been identified as a risk factor for malignant ventricular arrhythmias and SCD [26,28]. QT dispersion reflects spatial differences in myocardial recovery time. Previous studies have shown that an increase in QT dispersion >60ms is associated with the development of malignant ventricular arrhythmias [16] and SCD [9].

In large population-based studies including elderly subjects, the prevalence of an altered QTc interval has been estimated to be approximately 8% [11]. In contrast, the COPD patients in the current study showed a fourfold higher prevalence of an altered QTc interval compared to the general population. In a case-control study, investigating the risk of SCD in patients with coronary heart disease with and without prolonged QTc interval, the prevalence of prolonged QTc interval was 39% in the group with subsequent SCD [29]. This prevalence of altered QTc in SCD patients is comparable with the findings of the current study in COPD patients.

In a retrospective study including 162 chronic heart disease patients, the prevalence of increased QT dispersion >60 ms was 24% [30], which is similar to our observations in COPD patients (24%). These findings suggest a comparable prevalence of increased QT dispersion in patients with chronic heart disease and COPD patients.

There are some previous studies examining possible factors leading to an alteration in cardiac repolarization in COPD patients, however, the COPD patients in these studies [16,31-33] were free from comorbidities. Zulli et al. [31] found a significant univariate association between QT dispersion with FEV_1_% pred_._ and FVC% pred. respectively in COPD patients. However, in the multivariate analysis correcting for covariates, this association did not remain statistically significant. Yildiz et al. [16] compared 30 COPD patients with and without increased QT dispersion regarding possible factors influencing QT dispersion and found no independently associated factors. Similarly, in our study, only pack years of smoking and use of statins was statistically significantly associated with QT dispersion in the univariate analysis. However, after correcting for age, BMI, SaO_2_ and use of medication, none of the possible influencing risk factors were independently associated with QT dispersion.

None of the previously described studies [16,31] examined possible factors leading to an altered QTc interval in COPD patients. In the current study, age, pack years of smoking, Pocock risk score, respiratory variables, use of combined inhaled long-acting β-adrenergic and steroid and consumption of statins were significantly associated with a QTc >450ms in univariate analysis. Only SaO_2_ remained statistically significantly associated with QTc >450 ms after correction for covariates in the multivariate analysis. Thus, hypoxia may be a risk factor for a prolonged QTc interval. There is some evidence that hypoxia may prolong repolarization duration. In a study by Roche et al. [34], healthy subjects were exposed to normobaric hypoxic conditions and it was found that hypoxia significantly prolonged the QTc interval. In 12 COPD patients, Tirlapur et al. [35] observed that patients who had a low mean basal SaO_2_ (<80%) showed electrocardiographic changes such as a prolonged QTc interval. These findings are somewhat in contrast to the study by Sarubbi et al. [33], where QTc was measured in 15 hypoxemic/hypercapnic COPD patients before and after oxygen therapy and was not significantly reduced after 24 h of oxygen therapy. Thus it is still a matter of debate whether hypoxia affects cardiac repolarization and the underlying mechanism through which hypoxia may possibly influence cardiac repolarization remains currently unknown.

There is some evidence that autonomic neuropathy may results in prolonged cardiac repolarization in COPD patients. In a case-control study by Stewart et al. [32], 17 COPD patients with autonomic neuropathy were compared to 17 COPD patients without autonomic neuropathy. QTc was significantly longer in COPD patients with autonomic neuropathy. However, autonomic neuropathy was not measured in our study and thus we cannot directly compare the results of the study by Stewart et al. with our findings.

The current study has some limitations. We performed standard resting 12-lead ECG and not continuous 24-h ECG. Therefore it is not possible to evaluate the prevalence of alteration in cardiac repolarization both during a longer daytime period and during sleep. Furthermore, the cross-sectional study design does not allow to establish a causal relationship between altered cardiac repolarization in COPD patients and malignant ventricular arrhythmias and SCD, respectively. If prolonged cardiac repolarization promotes sudden cardiac death in COPD patients is currently unknown. Further controlled and longitudinal studies are needed to evaluate if prolonged cardiac repolarization promotes malignant ventricular arrhythmia and SCD in COPD patients.

## Conclusion

In conclusion, our findings suggest that one third of a typical COPD population has altered cardiac repolarization and increased dispersion of repolarization, which may be related to hypoxia. Altered cardiac repolarization may expose these patients to an increased risk for malignant ventricular arrhythmias and SCD.

## Competing interests

The authors declare that they have no competing interests.

## Authors’ contributions

NAS and MK had full access to all of the data in the study and take responsibility for the integrity of the data and the accuracy of the data analysis. NAS, CFC, GC, VAR and ARJVG contributed to the acquisition of data, data analysis and interpretation, drafting of the manuscript, revision for intellectual content and approved the final version. MK contributed to the conception and design, acquisition of data, data analysis and interpretation, drafting of the manuscript, revision for intellectual content and approved the final version. All authors read and approved the final manuscript.

## Pre-publication history

The pre-publication history for this paper can be accessed here:

http://www.biomedcentral.com/1471-2466/14/55/prepub
